# Slowing less than 1 Hz is decreased near the seizure onset zone

**DOI:** 10.1038/s41598-019-42347-y

**Published:** 2019-04-17

**Authors:** Brian Nils Lundstrom, Melanie Boly, Robert Duckrow, Hitten P. Zaveri, Hal Blumenfeld

**Affiliations:** 1 Mayo Clinic, Rochester, MN USA; 2 University of Wisconsin, Madison, WI USA; 3 Yale University, New Haven, CT USA

**Keywords:** Epilepsy, Excitability, Epilepsy

## Abstract

Focal slowing (<4 Hz) of brain waves is often associated with focal cerebral dysfunction and is assumed to be increased closest to the location of dysfunction. Prior work suggests that slowing may be comprised of at least two distinct neural mechanisms: slow oscillation activity (<1 Hz) may reflect primarily inhibitory cortical mechanisms while power in the delta frequency (1–4 Hz) may correlate with local synaptic strength. In focal epilepsy patients, we examined slow wave activity near and far from the seizure onset zone (SOZ) during wake, sleep, and postictal states using intracranial electroencephalography. We found that slow oscillation (0.3–1 Hz) activity was decreased near the SOZ, while delta activity (2–4 Hz) activity was increased. This finding was most prominent during sleep, and accompanied by a loss of long-range intra-hemispheric synchrony. In contrast to sleep, postictal slowing was characterized by a broadband increase of spectral power, and showed a reduced modulatory effect of slow oscillations on higher frequencies. These results suggest slow oscillation focal slowing is reduced near the seizure onset zone, perhaps reflecting reduced inhibitory activity. Dissociation between slow oscillation and delta slowing could help localize the seizure onset zone from interictal intracranial recordings.

## Introduction

Oscillatory brain waves recorded by electroencephalography (EEG) have been categorized into frequency bands spanning 1–50 Hz. More recently, it has been appreciated that broadband EEG (~0.01–500 Hz) contains richer cortical information^1^, with high-frequency oscillations receiving the most attention as markers of cognitive function and pathological tissue^2^. However, slow oscillations (<1 Hz) have been shown to modulate cortical excitability^3^ and during the pre-ictal period can localize the seizure onset zone^4^. Despite evidence linking slow oscillations to the regulation of neuronal excitability, slow frequency changes related to epileptic cortex remain less well studied.

The power spectral density quantifies the contribution of each frequency band to a signal, and for EEG signals lower frequency bands contribute much more power than higher frequencies^5^. Diffusely increased slow wave activity (SWA), approximately 0.1–4 Hz, is a hallmark of sleep^6^, while focal or global SWA changes can also be a sign of cerebral dysfunction^7^. The mechanisms underlying physiologic and pathophysiologic SWA remain unclear. Activity less than approximately 1 Hz may represent activated and quiescent neuronal states^8^ and function as the default activity of cortical circuits^9^. Increased power in frequency bands less than 1 Hz may reflect intrinsic neuronal mechanisms that dampen excitability^10–12^. On the other hand, activity that includes delta frequencies (1–4 Hz) during sleep is correlated with local synaptic strength and is regulated both globally, constituting a marker of sleep need, and locally^13–15^. Relative to slow oscillations, activity that includes delta power may be influenced to a greater extent by thalamic circuits^16,17^. Due to these potential differences in underlying mechanisms, we examine SWA with frequencies <1 Hz and SWA limited to higher frequencies in the delta power range.

Distinguishing physiologic from pathologic SWA remains difficult. In patients with seizures, delta activity in frontal and parietal areas is increased during seizures that impair awareness^18^. The spatial distribution of SWA during sleep and postictal states is generally considered diffuse, symmetric, and frontally predominant^19^, although iEEG data suggests a more heterogenous distribution^20^. Recent work in focal epilepsy suggests the location of SWA is related to the seizure onset zone (SOZ)^14^. During non-REM sleep, delta activity was increased for patients with focal epilepsy compared to controls both diffusely as well as locally with a maximal increase at the SOZ^14^. One difficulty in differentiating pathological from physiological slowing is that the much prior work comes from scalp EEG, which has a spatial resolution of about 10 cm^2^ of cortex^21^. Although iEEG offers the benefit of increased spatial resolution, usually coverage is predominantly or exclusively over one brain region, and, for example, the ability to compare the ipsilateral hemisphere to the contralateral hemisphere is absent. For the present analysis we selected a rare dataset of patients with extensive bilateral subdural EEG recordings.

Despite evidence suggesting distinct physiologic mechanisms underlying activity <1 Hz from faster SWA, it remains unclear whether these differences are evident at the macroscopic level relevant for clinical EEG. Given data supporting mechanistic differences in slow oscillation activity (<1 Hz)^6,8,10,16,22^ and activity that includes delta frequencies^16,17^, we hypothesized that these two power bands would differ with distance from the SOZ and between postictal slowing compared to sleep.

Changes in low frequency power are expected to alter spatial correlations^21^ and coupling with higher frequency activity^23^. There appears to be increased synchrony near the SOZ^24,25^ with overall decreased spatial synchrony for subjects with epilepsy compared to normal subjects^26^. Increased phase amplitude coupling between low frequencies (<4 Hz) and faster frequencies has been associated with increased cognitive performance in normal subjects^27^ and can be increased interictally near the SOZ^26,28^, suggesting cross-frequency coupling may be increased when cortical excitability is increased.

Here, we examine how interictal slow oscillation and delta activity is spatially distributed relative to the SOZ. We take advantage of a cohort of patients that had extensive subdural electrode coverage over both hemispheres, data becoming rarer with the increasing use of stereotactic depth electrode techniques^29^, and which allows us to sample an extensive region of cortex from ipsilateral and contralateral hemispheres. We also compare sleep and postictal states. We focus on postictal slowing immediately following focal seizure activity that did not lead to bilateral convulsive seizures, with the intent of comparing transient and prolonged slowing of focal origin, which is clinically often indistinguishable. Finally, we examine spatial correlations and cross frequency coupling relative to both the SOZ and behavioral state.

## Methods

### Patient data

This retrospective analysis with consent waiver was approved by the Institutional Review Board of Yale University. Data from patients undergoing intracranial EEG evaluation at Yale University from January 2014 to August 2017 were examined. Eight patients met the following inclusion criteria: (1) extensive bilateral subdural electrode coverage defined as at least 30 contacts per hemisphere, (2) no widespread abnormalities on MRI, and (3) well-defined seizure onset zones (SOZs). These criteria were chosen to ensure broad bilateral cortical coverage and to exclude cortex with any known widespread abnormalities that could affect slow wave activity. SOZs were determined at a multi-disciplinary epilepsy conference and were present in the left frontal (*n* = 3), right frontal (*n* = 1), left temporal (*n* = 1), right temporal (*n* = 4), and right parietal/occipital (*n* = 1) lobes. One patient had two right-sided SOZs in the temporal as well as parietal/occipital regions. Another patient likely had bilateral mesial temporal seizures, although seizure activity was arguably more prominent from the left side. For this patient, no definite interictal or ictal epileptiform activity was recorded from right-sided subdural contacts, and all seizure activity considered for this study (as detailed below) was left-sided. Thus, for this patient the left and right hemispheres were considered to be ipsilateral and contralateral to the SOZ, respectively. Patient data are summarized in Table 1.

**Table 1:** Abbreviations: amb = ambidextrous, AMTL = anteromesial temporal lobectomy, bilat = bilateral, DVA = developmental venous abnormality, F = female, FCD = focal cortical dysplasia, HC = hippocampus, L = left, M = male, OCD = obsessive compulsive disorder, R = right, RNS = Responsive NeuroStimulation.

Gender/Hand	Age/Onset Age	SOZ	MRI	PET	Outcome	Comments
M/R	30/17	L > R mesial temporal lobe	Non-lesional	Normal	Offered bitemporal RNS but declined; lost to follow-up	R-sided epileptiform activity recorded in depth but not subdural contacts
M/Amb	21/5	L orbitofrontal region	L inferior frontal FCD	L dorsolateral frontal hypometabolism	Resection with RNS; Engel 4a at 2 year follow-up	Pathology reactive astrocytosis
M/R	23/10	L orbitofrontal	L frontal DVA; L HC with poor internal architecture	Mild L inferior frontal hypometabolism	RNS, Engel 4 at 1 year	
F/R	48/17	R amygdala	L > R amygdala: increased fullness	R > L bilateral MTL hypometabolism	R AMTL, Engle 1B at 2 years	Pathology mild reactive astrocytosis
F/R	31/8	R anterior temporal and R occipital lobes	R occipital dysplasia	R temporal hypometabolism	Resection R occipital/temporal region with HC resection, Engel 1d at 1.5 years	Pathology with occipital 2b dysplasia, HC wnl
M/R	34/12	L posterior superior frontal gyrus	Non-lesional	Mild R temporal hypometabolism	No surgery, seizures twice monthly	Complications related to infection
F/R	31/9	R anterior temporal lobe	Non-lesional	R temporal hypometabolism	R AMTL, Engel 4 at 2 years	Pathology reactive gliosis
M/R	44/26	R superior mesial frontal region	Bilat anterior cingulotomies	Bilat medial frontal hypometabolism	Resection R superior/mesial frontal region, Engel 3a at 2.5 years	Bilat anterior cingulotomy 22 years prior for OCD; pathology reactive gliosis

### Intracranial EEG data

**Figure 1:**
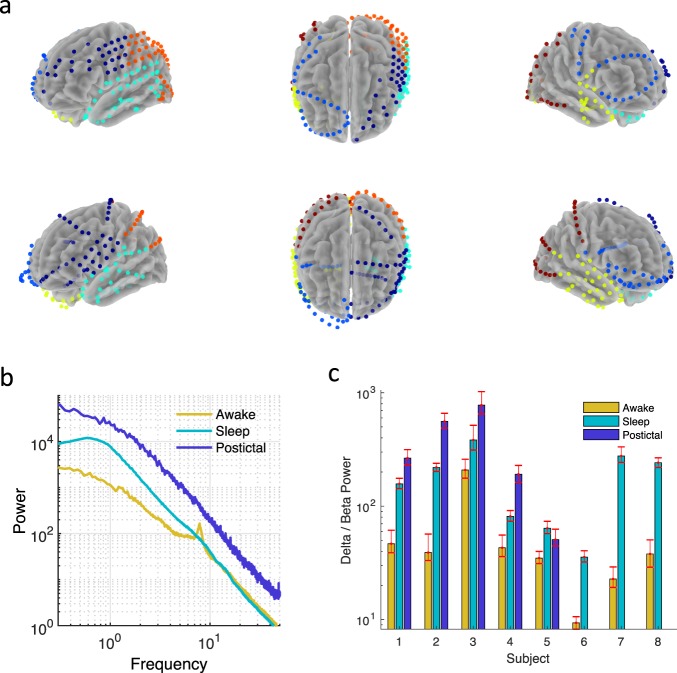
Sleep and Postictal states show increased slow wave activity. (**a**) Electrode placements registered to common coordinates for two example patients (each row). Colors represent segmentation by frontal (dark blue = L, light blue = R), temporal (cyan = L, yellow = R), and parietal/occipital (orange = L, red = right) lobes for each hemisphere. (**b**) Example power spectra averaged across all contacts from one subject. (**c**) Sleep and postictal states show increased delta (1–4 Hz) relative to beta (13–25 Hz) activity compared to the awake state. Power ratio was calculated for each contact and averaged across all contacts. Error bars represent 95% confidence intervals determined by bootstrapping.

Natus Neurolink IP 256 channel EEG amplifier (0.16 Hz high pass filter, 1024 Hz sampling frequency) was used to record intracranial EEG (iEEG) data from eight patients with 196, 172, 203, 201, 199, 213, 154, and 223 subdural contacts. 2, 4, 5, 4, 29, 12, 1, and 4 contacts for these patients were clinically determined to include the SOZ, respectively. To analyze contacts near the SOZ we used all contacts within two cm of these clinically determined SOZ contacts, which gave 7, 22, 11, 12, 66, 31, 11, and 16 contacts for the eight patients, respectively. Note that 97 (55%) contacts come from two of the eight patients. 1061 contacts were ipsilateral to the SOZ and 500 contralateral. Five patients had at least three focal seizures that did not progress to generalized convulsive activity. For these patients, 15-minute EEG epochs were taken during the awake, sleep, and postictal states following each of the first 3 recorded non-generalized seizures, for a total of nine epochs per patient. Awake and sleep epochs were visually inspected and chosen to avoid artifacts. Epochs for awake and sleep states were taken at least two hours following the seizure, while postictal state epochs were commenced immediately following the clinical determination of seizure offset. Sleep epochs were taken from approximately 11 pm to 3 am when there was widespread high amplitude (greater than 100 microvolts) slow wave activity over the frontal lobe intracranial contacts. Scalp electrode data were not sufficient for sleep scoring, and video was not available. Thus, for each patient we measured the ratio of delta (1–4 Hz) to beta (13–25 Hz) power, which is a widely accepted signature of non-REM sleep activity^30^, and found that this ratio was increased for all patients (Fig. 1c). If two seizures occurred during the day, then two sleep epochs were taken from the subsequent night. Awake epochs were taken during daytime hours when slow wave activity was clearly not present. Despite this, since video was not available, drowsiness or light sleep may have been present during awake states. For the remaining three patients who did not have at least three focal seizures without generalized convulsions, three epochs of awake and sleep activity, for a total of 6 epochs per patient, were recorded from the 24-hour period prior to their first seizure.

### Data processing and power spectrum analyses

Data were low pass filtered with an 8^th^ order Chebyshev Type I filter (cutoff frequency 102.4 Hz) prior to downsampling to 256 Hz for data manipulation. Post-implantation imaging was coregistered to the standard MNI space using BioImage Suite. Locations were determined for each contact and manually segmented into six regions: frontal, temporal and parietal/occipital regions for each side. Contacts involved in seizure onset were determined from clinical reports. Data for each contact were filtered with zero-phase high and low pass fourth-order butterworth filters with cutoff frequencies of 0.1 and 100 Hz, respectively. Frequencies between 0.3 Hz and 50 Hz were considered following computation of the power spectral density by Welch’s method. Spectra were normalized by mean total power from 0.3 to 50 Hz to avoid undue influence specific to experimental conditions of the contact (e.g. impedance differences, CSF shunting). Power spectra for each contact were calculated and normalized. These estimates were averaged across contacts for three groups: (1) contacts that were ipsilateral to the SOZ and within 2 cm of clinically defined SOZ contacts, (2) contacts that were ipsilateral and more than 2 cm away, and (3) contacts that were contralateral to the SOZ. Power spectra from each contact and epoch across patients were averaged according to these three groups, except as otherwise noted.

Power bands were chosen according to typical clinical definitions^7^ and prior work^26^ to include slow oscillation (0.3–1 Hz), delta (2–4 Hz), theta and alpha (4–13 Hz), and high beta and gamma (20–50 Hz) frequencies. When comparing slow oscillation and delta powers, we limited delta power to 2–4 Hz given the arbitrary nature of frequency band definitions and evidence of 1–2 Hz as a transition zone.

### Correlations between contacts and cross-frequency coupling

Correlations can be assessed spatially between contacts and between frequency bands within a single contact, also known as cross-frequency coupling. For spatial correlations, the Euclidean distance was calculated between all contacts. To assess the strength of the linear relation relationship between contacts, a single Pearson correlation coefficient without any time lag was calculated for each contact pair for all contact combinations. The Fisher z-transformation was used to stabilize the variances of the Pearson coefficients prior to averaging.

Data from the 15-minute epochs for Awake, Sleep, and Postictal states were bandpass filtered with fourth-order Butterworth filters, and the correlation coefficients calculated.

To assess correlations between frequency bands, i.e. cross-frequency coupling, we used the Synchronization Index (SI)^31^ to measure the synchrony between the phase of a slower frequency band (0.3–1 Hz) and the power envelope of a higher frequency band (20–50 Hz). Phases *θ*_*L*_ and *θ*_*H*_ were obtained for the slow frequency band (0.3–1 Hz) and for the envelope of higher frequency activity, respectively. To find the phase *θ*_*L*_, data were bandpass filtered between 0.3–1 Hz. We used the Hilbert transform to obtain the discrete-time analytic signal. The instantaneous phase of the dominant frequency within the passband of the signal was determined from the analytic signal. To find the phase *θ*_*H*_, data were bandpass filtered, and the time-varying envelope of the signal power was obtained as the squared magnitude of the discrete-time analytic signal. The instantaneous phase *θ*_*H*_ is then found as the phase of the Hilbert transform as above. SI is defined to be $\,{\sum }^{}{e}^{i({\theta }_{L}-{\theta }_{H})}/N$, where *N* is number of time points and *θ* is the instantaneous phase. The magnitude of SI reflects the extent to which *θ*_*L*_ and *θ*_*H*_ are synchronized. It is calculated for each contact, is equivalent to one minus the circular variance of the phase difference *θ*_*L*_ − *θ*_*H*_^32^, and is a measure of the concentration of the phase differences. As |SI| increases, the dispersion and circular variance of the phase difference decreases. The phase of SI reflects the “preferred synchronization phase”, the phase of *θ*_*L*_ for which coupling is maximal. A circular average is used to find the mean phase.

### Statistical analysis

95% confidence intervals (bias corrected and accelerated) were obtained by bootstrapping (1,000 to 10,000 iterations), which is a widely accepted statistical resampling technique that relies on random sampling with replacement of the distribution in question to provide a non-parametric and robust confidence interval estimate^33^. To obtain confidence intervals, the variance for distribution *N* with *n* values is estimated by creating *k* bootstrapped distributions from distribution *N*. Each of the *k* distributions is created by randomly drawing *n* samples from distribution *N* with replacement. Thus, more frequent values in distribution *N* have a greater representation in the *k* bootstrapped distributions, allowing for a non-parametric estimate of variance to be calculated using accepted formulas, such as via the “bootci” function in Matlab (Mathworks, 2013). To minimize false positive results due to multiple comparisons, we limited our analyses to those motivated by initial hypotheses and evaluated pooled data prior to individual data. Correction for multiple comparisons was not performed for non-pooled data. Non-overlapping error bars indicate that the population means may be considered significantly different with an error of less than 5%, or *p* < 0.05, as is typical for hypothesis testing. A two-sample *t*-test was used for hypothesis testing as reported in Results.

All data and analyses are available upon request.

## Results

**Figure 2:**
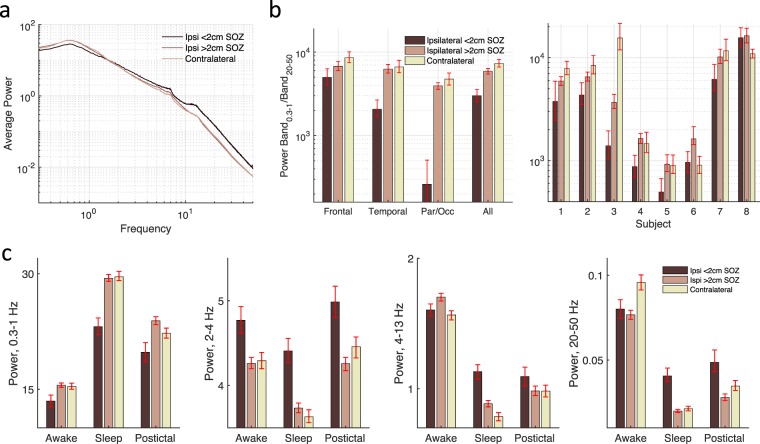
Decreased power <1 Hz near the SOZ. (**a**) Average power for contacts stratified by their relation to the SOZ during sleep. (**b**) Power ratio of 0.3–1 Hz band to 20–50 Hz band for all contacts across subjects for frontal, temporal, and parietal/occipital contacts (left panel) as well as per patient (right panel) during sleep. Power <1 Hz during sleep is significantly decreased near the SOZ for six of eight patients. (**c**) Power by state for four frequency bands. Power is normalized by the average power from 0.3–50 Hz for each contact in the respective state. Error bars represent 95% confidence intervals determined by bootstrapping.

The locations of 1561 electrodes from 8 patients (mean 28.9 years, range 17–44) were determined in standard MNI space and manually segmented into frontal, temporal and parietal/occipital head regions (Fig. 1a). Given its association with SWA, we examined first the non-rapid eye movement sleep state. Sleep states showed increased ratio of delta to beta power compared to the awake state for all eight patients (*p* < 0.05, Fig. 1b,c), in agreement with prior work classifying intracranial data into behavioral states^30^. Average power spectra showed decreased slow oscillation power, a transition band from approximately 1–2 Hz, and increased higher frequency power for contacts near the SOZ (Fig. 2a). To quantify this tilt of the power spectra, we examined the ratio of slow to fast activity. The decreased ratio of slow oscillation (0.3–1 Hz) to high beta and low gamma (20–50 Hz) activity was evident for SOZs located in any head region, and at the subject level for six of eight subjects (Fig. 2b). For the contacts closest to the SOZ, relative power in the 0.3–1 Hz frequency band was significantly decreased by 11–22% as compared to power in the same band in contralateral contacts (Fig. 2c). Specifically, power was decreased most prominently in the sleep state by 22% (t(1939) = −10–5, *p* < 1e-24), compared with decrements of 14% for the awake state (t(1939) = −4.6, *p* < 1e-5) and 11% for the postictal state (t(1255) = −3.6, *p* < 1e-3). In contrast, relative power in the 2–4 Hz frequency band was significantly increased by 10–18% for contacts closest to the SOZ. Specifically, power was increased by 10% for the awake state (t(1939) = 5.2, *p* < 1e-6), by 18% for the sleep state (t(1939) = 10.2, *p* < 1e-23), and 11% for the postictal state (t(1255) = 4.6, *p* < 1e-6). Similarly, there was increased relative activity in the 20–50 Hz frequency band for the sleep (t(1939) = 11.7, *p* < 1e-29) and postictal states (t(1255) = 4.4, *p* < 1e-4) postictal states. Increased high frequency power is also seen within the SOZ in the 4–13 Hz band during sleep but not awake and postictal states. Increased power in the 20–50 Hz band was not present in the SOZ during the awake state compared to sleep, perhaps representing cortical dysfunction.

**Figure 3:**
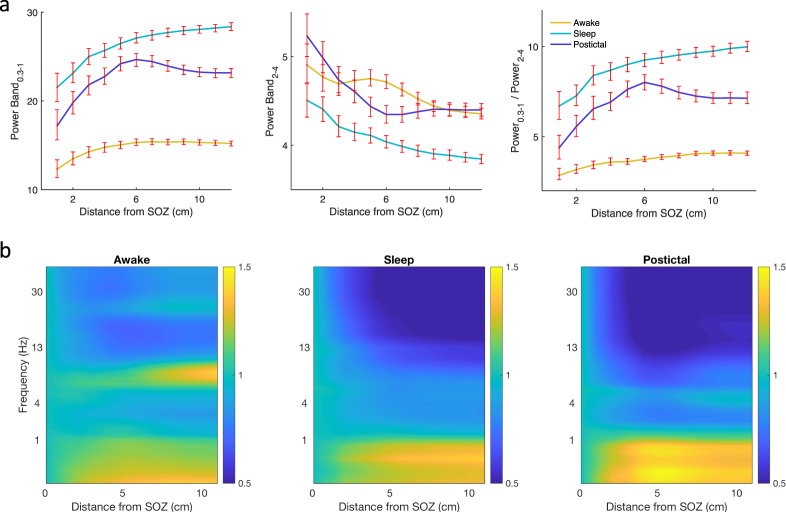
Relative slow oscillation power increases with distance from the SOZ. (**a**) Power band 0.3–1 Hz increases with distance from the SOZ for contacts ipsilateral to the SOZ, while power in the 2–4 Hz band decreases. (**b**) Change in power relative to power in contacts less than 1 cm from the SOZ. All contacts within a given distance from the SOZ are included. Error bars represent 95% confidence intervals determined by bootstrapping.

We expected slow wave power to vary as a function of distance from the SOZ and wondered whether slow oscillation power would vary differently than delta power given the possibly different mechanisms underlying activity in these bands. Given the apparent transition band for 1–2 Hz power in Figs 2a and 3b, we considered power bands of 0.3–1 Hz and 2–4 Hz for slow oscillation and delta activity, respectively. For a given distance from the SOZ, all contacts within that distance to the nearest SOZ contact were included. Power for slow oscillations increased with distance from the SOZ, while power for delta activity decreased (Fig. 3). This was true in the awake, sleep, and postictal states. Specifically, when compared to contacts 12 cm from the SOZ, contacts within 1 cm of the SOZ showed increased slow oscillation power by 23% for the awake state (t(3454) = 5.59, *p* < 1e-7), 32% for the sleep state (t(3454) = 8.8, *p* < 1e-17), and 35% for the postictal state (t(2131) = 6.8, P < 1e-10). In contrast, the same contacts showed relatively decreased delta activity by 11% for the awake state (t(3454) = −5.0, *p* < 1e-6), 15% for the sleep state (t(3454) = −6.7, *p* < 1e-10), and 16% for the postictal state (t(2131) = −6.5, *p* < 1e-10).

**Figure 4:**
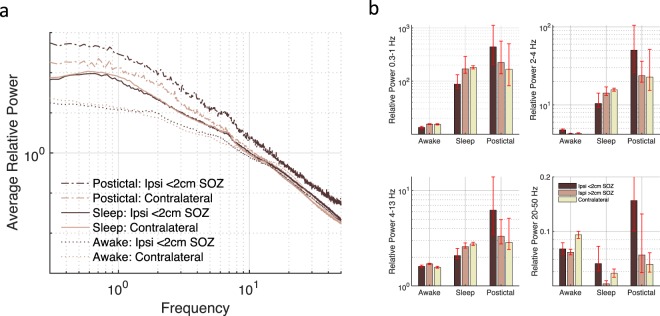
Broadband power is increased during the postictal state. (**a**) Average spectra near and contralateral to the SOZ for awake, sleep, and postictal states. (**b**) Power bands corresponding to the power spectra. Power is relative to the mean awake power (0.3–50 Hz) for each contact.

We compared postictal slowing to sleep, given that they represent different clinical states but can appear similar on EEG during visual inspection. Contacts near the SOZ show a broadband increase during postictal slowing compared to the awake state (Fig. 4). In contrast, for the sleep state contacts were increased relative to the awake state for low but not high frequencies. Compared to sleep, the ratio of slow oscillation to delta activity during the postictal state is reduced by 35% (t(454) = −5.8, P < 1e-4) for ipsilateral contacts within 2 cm of a SOZ and by 29% (t(5131) = −12.9, P < 1e-36) for contacts within 12 cm of a SOZ (Fig. 3a, right panel).

**Figure 5:**
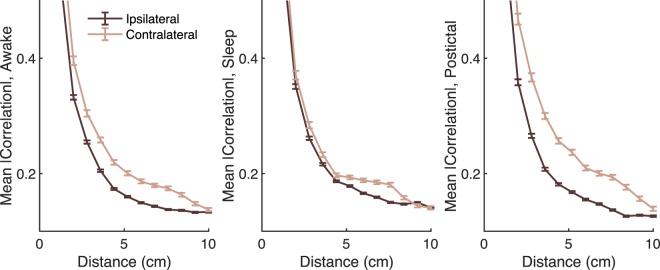
Correlations for the 0.3–1 Hz frequency band are increased for the contralateral hemisphere, shown during sleep. Correlations are Fisher z-transformed prior to averaging. Error bars represent 95% confidence intervals determined by bootstrapping.

We examined spatial correlations and synchrony for SWA with the hypotheses that spatial synchrony would be reduced ipsilateral to the SOZ and that cross-frequency coupling would be increased within the SOZ. Spatial correlations for SWA were reduced for contacts ipsilateral to the SOZ, shown for 0.3–1 Hz activity in the awake, sleep and postictal states (Fig. 5). Similar differences were seen for 2–4 Hz activity but not higher frequency bands. The correlation differences were most robust in the frontal lobes (data not shown).

**Figure 6:**
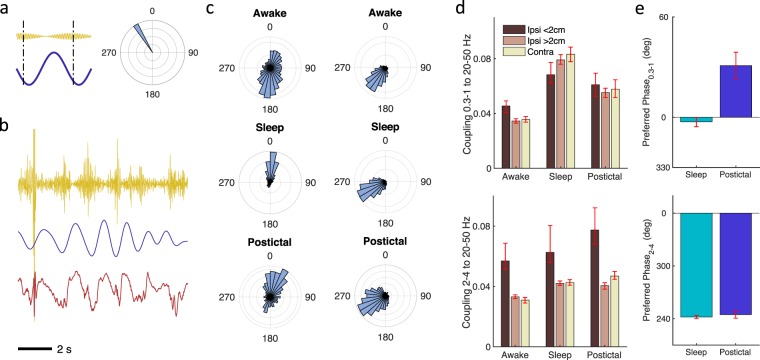
Cross frequency coupling between slow frequencies and 20–50 Hz activity. (**a**) Example showing maximal coupling of 330 degrees between slow and high frequencies (dashed vertical lines, left panel) displayed on a polar plot (right panel). Zero degrees represents the peak of the surface positive slow frequency (positive is downward). (**b**) Example data from a single channel during sleep showing raw data (bottom row), 0.3–1 Hz filtered data (middle row), and 20–50 Hz filtered data. (**c**) Circular histograms of the preferred slow frequency coupling phase for each electrode channel for the 0.3–1 Hz (left column) and 2–4 Hz (right column) frequency bands to 20–50 Hz activity. (**d**) Coupling strength between the 0.3–1 Hz (upper panel) or 2–4 Hz (lower panel) and 20–50 Hz activity. Coupling strength is inversely related to the spread seen in the circular histograms. The darkest bar represents ipsilateral contacts <2 cm from the SOZ, the middle bar ipsilateral contacts >2 cm from the SOZ, and the lightest bar contralateral contacts. (**e**) Preferred coupling phase of the slow wave activity for the sleep and postictal state. Error bars represent 95% confidence intervals determined by bootstrapping.

Finally, we examined synchrony between frequency bands (Fig. 6a,b) via phase amplitude coupling assessed via a Synchronization Index^31^, and focused on SWA as the underlying rhythm^3,16^. Maximal coupling to 20–50 Hz activity occurs around the positive peak of the 0.3–1 Hz activity (Fig. 6c, left column), consistent with prior data showing that slow oscillations modulate higher frequency activity during sleep^3,34^. Periods of increased activity, or “up” states, often occur during the surface positive portion of the slow wave oscillation^6,35^. Slow oscillation coupling during the postictal state is generally reduced compared to sleep (Fig. 6d, top row), and occurs later during the slow oscillation phase after the up state peak. Together with Fig. 3, these results suggest slow oscillations have a weaker modulatory effect during postictal than sleep states. Activity in the 2–4 Hz band is a relatively stronger modulator of high frequency activity near the SOZ (Fig. 6d, bottom row).

## Discussion

Slow wave activity (SWA) has long been associated with sleep and postictal states. Here, we find that slow oscillation activity (0.3–1 Hz) is decreased near the seizure onset zone (SOZ), while delta activity (2–4 Hz) is increased, suggesting that these frequency bands may result from different underlying mechanisms. We also show that postictal slowing is comprised of broadband power increases and has distinct characteristics from sleep.

### Underlying physiology of normal and pathological slow waves

Slow oscillations at approximately 0.3–1 Hz modulate faster frequencies^3,35,36^ with periods of activity alternating between “up” and “down” states^6^. Layer 5 cortical neurons probably initiate up states, which persist due to recurrent excitatory synaptic activity^8^; up states are terminated either due to increased inhibitory inputs^22^ or as a result of activity-dependent potassium conductances^8^. Activity-dependent potassium conductances are associated with regulation of neuronal activity and action potential firing rate adaptation^37^. When inhibitory inputs are decreased, it appears that activity-dependent potassium conductances exert a relatively stronger dampening effect and decrease slow oscillation frequency^12^. These potassium conductances may be reduced near the SOZ, which could lead to decreased slow oscillation power^10,11^. Many antiepileptic medications effectively increase firing rate adaptation via sodium-^38^ or potassium-related^39^ mechanisms, suggesting that these medications could alter low frequency power spectra of patients. Other data show that slow wave power including delta activity may reflect the strength of synaptic inputs^13,14^, where delta activity has been associated with thalamocortical inputs^36^ and lower frequency activity persisted without thalamic input^17^. Interictal epileptiform activity could contribute to SWA, as spike shape and frequency changes near the SOZ^26,40^, and interictal epileptiform activity contains a wide range of frequencies including delta and beta activity^41,42^. Overall, recent data support the notion that activity in the 0.3 to 4 Hz range likely represents at least two distinct processes^43^.

### Slow waves in sleep and postictal states

Non-rapid eye movement sleep especially occurring during the first portion of the night have long been associated with increased slow wave activity^6^. Sleep deprivation results in a decreased number of slow waves below 1 Hz and increased EEG power greater than 1 Hz^44^. Recent work has compared delta activity during slow wave sleep from normal individuals and those with epilepsy^14^, finding increased activity near the SOZ. Increased 1–4 Hz activity was also correlated with increased seizure activity over the previous 3–5 days and increased interictal epileptiform activity the hour prior to bedtime. One interpretation is that the SOZ and epileptiform activity represent increased neuronal activity that requires increased synaptic renormalization, which leads to increased delta activity during sleep^13^. These results are consistent with the idea that the SOZ represents a region of increased neuronal activity reflected in reduced 0.3–1 Hz activity, which could reflect reduced potassium conductances^10,11^. Postictal slowing may represent increased excitability relative to the slowing of sleep, given its reduced ratio of slow oscillation to delta power. The broadband increases seen during postictal states are consistent with an overall increased number of neural units contributing to the spectral power^5^. Thus, what is referred to as postictal slowing may be an increased number of active asynchronous neurons, rather than a smaller group of synchronous neurons as for sleep.

### Dissociation between slow oscillation and delta activity may localize the SOZ

Changes in the composition of slow wave power can appear on the centimeter spatial scale^15^ and could be useful for the localization of the SOZ. Prior work focusing on lower frequencies (<0.5 Hz) described the utility of DC shifts recorded just prior to seizures to localize the SOZ^4,45^. This infraslow activity is often present prior to seizures^46^ and may have non-neuronal physiological mechanisms^47^. The reduced slow activity described here is somewhat faster and appears to be a static property of the SOZ, most notable during sleep and in conjunction with increased activity in the beta and low gamma frequency bands. Similar decreases in slow activity and increases in fast activity during event-related tasks have been associated with activated cortex^48^, and may be related to local desynchronization with a relative increase of excitatory drive, which may also be found in epileptogenic cortex.

### Interictal spatial and temporal synchrony

Prior work has examined synchrony from subdural iEEG data from epileptic cortex. Linear spatial correlations were increased within the SOZ^25^ and decreased between electrodes that bridge the SOZ and non-SOZ^24^, consistent with the idea that the SOZ represents an area of increased local synchrony. Despite this, long-range spatial correlations remain^25^. Here, spatial coupling is reduced in the hemisphere ipsilateral to the SOZ, consistent with the notion that the SOZ disrupts long-range spatial synchrony in the ipsilateral hemisphere. Phase amplitude coupling has been noted to be increased within the SOZ^26,28^. Specifically, during sleep the preferred coupling phase for 0.3–4 Hz to 30–80 Hz activity was noted to occur roughly at the down to up state transition (about 270 degrees)^28^. Here, slow oscillation activity had a preferred coupling phase of approximately 0 degrees, while delta activity had a phase of approximately 260. Increased coupling and an earlier phase may be related to more excitable cortex^26,28,49,50^. Especially during the postictal state, delta rather than slow oscillation activity may have an increased modulatory role near the SOZ: power in the slow oscillation frequency range (0.3–1 Hz) is decreased in the SOZ, cross-frequency coupling is increased near the SOZ for the 2–4 Hz power band, and the preferred coupling phase for 2–4 Hz activity occurs earlier than the 0.3–1 Hz activity.

### Limitations

This study is limited by the inclusion of eight patients, five of whom had repeated focal seizures that allowed for the analysis of the postictal state. Further, patient specific electrode coverage limits subject level conclusions. For example, differences in SWA may be more prominent for certain head regions or epilepsy types. More than 1500 subdural electrodes were implanted allowing for conclusions to be drawn from group analysis of contacts. Nonetheless conclusions from our results are limited by the non-uniformity of the electrode coverage between patients. For example, 55% of the contacts that were less than 2 cm from the SOZ arose from two of the eight patients. To address this non-uniformity, we analyzed our results in two ways: by averaging data for each patient to assess inter-patient differences (e.g. Fig. 2b right panel), and by averaging across all electrodes of a given type to avoid weighting some channels more than others (e.g. Fig. 2b left panel). We were limited by the 0.16 Hz cutoff frequency of the amplifier hardware high pass filter, which gives nonlinear phase shifts below the cutoff frequency. Hence, we examined frequencies greater than 0.3 Hz. Although sleep was not formally staged due to the absence of appropriate scalp data, during the majority of epochs iEEG showed a clear state change marked by high amplitude slow wave activity and increased ratio of delta to beta power, which are widely accepted signatures of non-REM sleep activity^30^.
